# Understanding the effectiveness and quality of virtual cancer multidisciplinary team meetings (MDTMs): a systematic scoping review

**DOI:** 10.1186/s12913-024-11984-z

**Published:** 2024-11-27

**Authors:** Anjola Onifade, Samantha L. Quaife, David Holden, Donna Chung, Martin Birchall, Michael D. Peake, Muntzer Mughal, Daisy McInnerney

**Affiliations:** 1 University Hospitals Sussex NHS Foundation Trust, Brighton, Sussex UK; 2 Centre for Cancer Screening, Prevention, and Early Diagnosis, Wolfson Institute of Population Health, Queen Mary University of London, London, UK; 3 University College London Hospitals NHS Foundation Trust, London, UK; 4 North Central London Cancer Alliance, London, UK; 5 Mid and South Essex NHS Foundation Trust, Basildon, Essex UK; 6 University of Leicester, Leicester, UK; 7 Cancer Research, London, UK

**Keywords:** Cancer, Virtual, Hybrid, Multidisciplinary team meetings, Quality, Effectiveness, Health service

## Abstract

**Introduction:**

Cancer multi-disciplinary team meetings (MDTM) assemble clinical experts to make diagnostic and treatment recommendations. MDTMs can take place in person, virtually, or in a hybrid format. Virtual and hybrid MDTMs have been in use for over two decades. This systematic scoping review aims to map the evidence on virtual and hybrid MDTM formats over time, providing insights into their quality, and the facilitators and barriers to their effective delivery.

**Methods:**

The PRISMA scoping review checklist has been followed. A systematic search of PubMed, PsychINFO, and Embase between 1990–2023 identified 9399 records. These were independently screened by two researchers to identify primary research of any design that assessed quality or effectiveness of cancer VMDTMs. Results were narratively synthesised.

**Results:**

Eight quantitative, two qualitative and three mixed-methods studies were included. All were observational and most were retrospective (*n* = 8). Varied outcome measures were used to evaluate meeting quality, including treatment recommendations, survival, time from diagnosis, and overall attendance. VMDTMs were superior (*N* = 6) or sometimes equivalent (*N* = 4) to face-to-face meetings. Studies identified implementation factors critical to their effective delivery, including internet-stability and chairing.

**Conclusion:**

The heterogeneous literature suggests VMDTMs offer some benefits over face-to-face meetings. Training and infrastructure are key to prevent risks to patient safety. A definitive comparative evaluation is needed to inform best practice.

**Supplementary Information:**

The online version contains supplementary material available at 10.1186/s12913-024-11984-z.

## Introduction

Multi-disciplinary team meetings (MDTMs) involve professionals from mixed clinical disciplines who collaborate to make recommendations about optimal care for patients [1]. They were first introduced in the 1990s as a vital component of cancer care to ensure equality of access to standardised and high-quality care [2–4]. This led to the subsequent requirement, outlined in the National Cancer Plan (2000), for a comprehensive assessment of each patient’s care by an MDT. MDTs enhance patient satisfaction, timeliness of treatment, and survival rates [5–8]. Consequently, MDTs have been introduced as part of the care pathway for other specialities, with many hospitals adopting regular MDTMs for chronic conditions [9–11].

MDTMs can take place in person, virtually through videoconferencing or telemedicine platforms, or in a hybrid format combining both methods. Virtual and hybrid MDTMs have been in practice for over two decades [12–14], primarily aiming to facilitate discussions on rare or unusual tumours and address logistical challenges in remote areas with limited healthcare resources [12]. However, during the COVID-19 pandemic, social distancing measures forced many MDTs to rapidly transition from face-to-face to virtual meetings and many have since continued to operate remotely. While virtual and hybrid MDTMs offer advantages such as overcoming geographical barriers, they also present distinct challenges like connectivity issues, technological difficulties, and potential distractions [15].

Given their widespread use, understanding the potential clinical impact of running MDTMs virtually is crucial. Existing individual studies have reported mixed findings. Virtual and hybrid MDTMs have shown promise in significantly improving decision-making about cancer patient care, by promoting interdisciplinary communication, and fostering educational opportunities across various levels of the clinical care team [16]. Tan and colleagues’ (2021) review of healthcare professionals’ (HCPs) experiences of telemedicine more broadly highlighted its potential to enable delivery of higher-quality care by improving communication and facilitating shared decision-making with patients, and families [17].

However, opinions are mixed regarding the user-friendliness of telemedicine and management of technical issues, both of which can hinder attendees’ ability to participate effectively in VMDTMs. Indeed, technical challenges were a substantial barrier during the initial integration phase of virtual meeting platforms into cancer MDTs, generating scepticism and reduced satisfaction among users [18, 19]. Another study emphasised the need for additional skills to effectively incorporate videoconferencing into MDTMs and ensure their intended goals are achieved [20].

Several original research papers have begun to explore the effectiveness and quality of virtual and/or hybrid cancer MDTMs. However, to date no systematic review has integrated their findings to form conclusions about their impact on MDTM quality and effectiveness. Indeed, it is difficult to answer specific questions about their quality and effectiveness compared to face-to-face meetings due to variable definitions of virtual and hybrid MDTMs, and heterogeneity of research methods used to evaluate MDTM quality and effectiveness. Given these challenges, we have conducted a scoping review to systematically explore and characterise the diverse range of existing literature. Scoping reviews enable the clarification of intricate concepts in a robust and reproducible manner, making it a suitable approach to organise, synthesise, and structure the existing body of research investigating MDT effectiveness and quality [21–23].

While a previous scoping review focused on participants' perspectives and limitations of virtual and hybrid MDTMs [24]. Our review seeks to extend this focus with a broader methodological lens. We aim to explore a wider array of studies evaluating MDTM quality and effectiveness, not just through participant experience but also via the use of observational tools, real-time assessments, and comprehensive methodological evaluations. This approach offers a more nuanced understanding of operational dynamics, potential efficiencies, and challenges. The findings will guide improvements in evaluation methods, identify avenues for further research, and highlight key factors that impact VMDTM delivery.

### Objectives

1. Primary objective:
  To map the existing literature evaluating the quality and effectiveness of virtual and hybrid cancer MDTMs.
2. Secondary objectives:
  To summarise definitions of virtual and hybrid cancer MDTMs.
  To categorise study designs and outcome measures used for MDTM evaluation.
  To describe and summarise the findings of studies reporting on the quality and effectiveness of virtual and hybrid cancer MDTMs.
  To identify facilitators and barriers to effective virtual and hybrid MDTMs implementation and delivery.

## Methods

This systematic scoping review follows the Arksey and O’Malley framework, Levac and colleagues^’^ extension, and the Joanna Briggs Institute guidelines [22, 25, 26]. The review process consists of six stages:

1. Research question identification.
2. Identification of relevant studies.
3. Study selection.
4. Data charting.
5. Collation, summarisation and reporting the results.
6. Consultation.

The review adheres to Preferred Reporting Items for Systematic Reviews and Meta-Analyses for Protocols and Scoping Reviews (PRISMA-ScR) guidelines [27] (Supplementary File 1) and was pre-registered on the Open Science Framework [28].

### Stage 1: Identifying the research question

To meet the aims of the review, the following research questions were defined:

- What evidence exists on the effectiveness and quality of virtual and hybrid cancer MDTMs?
- How are virtual and hybrid MDTMs defined in studies?
- What facilitators and barriers impact the implementation and delivery of virtual and hybrid MDTMs?

### Stage 2: Identifying relevant studies

#### Eligibility criteria

Inclusion criteria

- Primary studies evaluating the quality and/or effectiveness of virtual or hybrid MDTMs to manage cancer patient care in any healthcare setting.
- All types of original research from the peer-reviewed medical and nursing, psychological and social science literature, including but not limited to: randomised controlled trials (RCTs), comparative studies (e.g. non-randomised experiments, before-and-after studies), qualitative studies, case studies, ethnographies, and diary studies.

Exclusion criteria:

- Studies not published in English language.
- Review articles
- Non-peer reviewed sources (e.g. book chapters, conference abstracts, and dissertations/theses).
- Studies published before 1990 (as MDTMs were introduced after that time).

The following preliminary definitions of virtual and hybrid MDTMs were used to guide study selection, based on existing literature [29]:

- MDTMs: a group of health and care staff who are members of different professions (e.g. general practitioners, social workers, nurses) and/or organisations collaborating to make decisions regarding individual patient and service user treatment.
- VMDTMs: all members join from different locations, facilitated by any mechanism (e.g. telephone, videoconferencing), or explicitly called virtual.
- Hybrid MDTMs: one or more members join from different locations, facilitated by any mechanism (e.g. telephone, videoconferencing), or explicitly called hybrid [30].

#### Databases and search strategy

We searched the databases Pubmed, PyschINFO and Embase databases on 21 January 2022 and again on 20 June 2023 before the final analysis to identify any newly published papers.

The search strategy was developed using the SPIDER (Sample, Phenomenon of Interest, Design, Evaluation, Research type) tool [31] (see Supplementary File 2).

### Stage 3: Study selection

A seven-stage screening process was followed. At each stage, eligibility criteria were iteratively discussed and updated for clarity and to ensure alignment, as per standard scoping review best-practice [26]:

1. Two reviewers (A.O and D.M) independently screened the title and abstracts of the first 100 search results for eligibility.
2. Reviewers compared and discussed their decisions to ensure alignment in the interpretation of the eligibility criteria.
3. Once alignment was achieved, the two reviewers independently screened the title and abstracts of all remaining search results.
4. Reviewers compared and discussed their decisions, resolving disagreements through discussion and consensus with the senior researcher (S.Q).
5. Full-text articles of all papers included in the title and abstract screening stage were independently screened for eligibility by the two reviewers.
6. Reference lists of included or other relevant studies and reviews identified through the database search were manually screened for relevant papers.

Reviewers compared and discussed their decisions, resolving disagreements through discussion and consensus with the senior researcher.

### Stage 4: Charting the data

A data charting and extraction framework (Supplementary File 3) was developed by two reviewers (A.O and D.M) to systematically guide data extraction from eligible papers across three categories: study characteristics, MDT characteristics and MDT effectiveness and quality. The framework was piloted on the first two studies to ensure its adequacy, and no modifications were needed thereafter.

Data extraction was conducted independently by reviewers (A.O and D.M), with a subset reviewed by the senior researcher. Disagreements were resolved through consensus and discussion with the senior researcher. Facilitators and barriers to effective MDT delivery were extracted from both qualitative primary data reported by studies and authors’ informal reflections in study discussion sections.

### Stage 5: Collation, summarisation and reporting of the results

A narrative synthesis approach was adopted to combine insights from all included studies, sequentially addressing each research question and summarising the reported quality and effectiveness of virtual and hybrid MDTMs [32]. This was supported by descriptive summary statistics, including the number and proportion of different study designs; the types of outcome measures used; and the reported effectiveness and quality of meetings.

### Stage 6: Consultation

The study management group (SMG) comprised clinicians with experience participating in cancer MDTMs and conducting MDT quality improvement exercises, behavioural scientists, NHS management professionals with MDT-specific expertise, and a patient and public involvement representative. The SMG was involved in the review’s design, oversaw the review’s conduct and progress, and contributed to the interpretation and write-up of results.

## Results

### Study selection

**Fig. 1:**
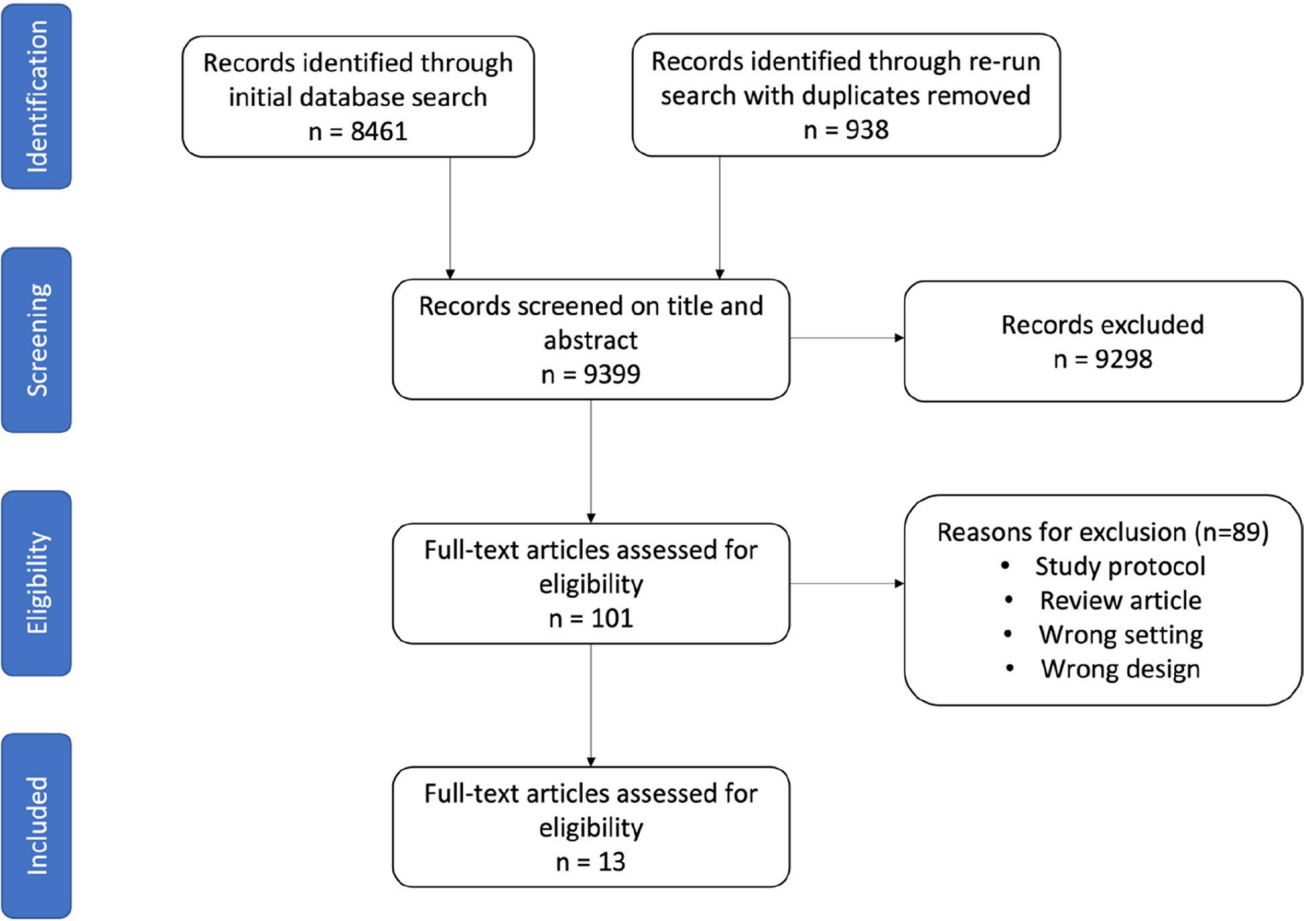
PRISMA diagram

The initial search yielded 8,461 unique results, and a subsequent re-run identified an additional 938 articles. After title and abstract screening, 101 full-texts were evaluated, and ultimately, 13 studies met the eligibility criteria (Fig. 1).

### Study characteristics, designs, and outcome measures

#### Overview of study characteristics: settings and designs

Detailed study characteristics are reported in Table 1. The included studies were conducted between 2014 to 2023, primarily in high-income countries: six in the United States [16, 33–37], two in Sweden [38, 39], two in Australia [40, 41], one in Italy [42], one in Canada [43] and one in United Kingdom [44]. Two studies [37, 43] involved teleconference meetings between MDT members from different countries. Of these, one [37] conducted clinicopathologic teleconferencing involving clinicians and pathologists from Malawi and the United States. The other [43] was part of a twinning neuro-oncology initiative between cancer hospitals in Jordan and Canada. Among the studies, eight [16, 34–37, 40, 43, 44] focused on a single cancer type (covering sarcoma [34], lymphoma [16], head and neck (H&N) [36], childhood central nervous system [43], lymphoproliferative disorders [37], upper gastrointestinal (GI) [40], and lung [41, 44], while the rest examined MDT efficacy across multiple cancer types. No key differences were noted between studies conducted pre and post COVID-19.

**Table 1:** Summary of studies assessing the quality and effectiveness of virtual and hybrid cancer MDTMs

Study	Design	Aims	Setting	Participants	Primary Outcomes	Secondary Outcomes
Groothuizen 2023 [32]	Mixed methods, Quantitative & Qualitative	Evaluate the impact of shift to virtual lung cancer MDTMs in response to COVID-19, in relation to size of information technology issues, distractions and MDT members’ perceptions and experiences of shift	Eight hospital organisations in Southern England	190 team members and managers	Continuity & efficiency of MDTs, number of members attending and cases reviewed, Quality of imaging and histo-pathology, satisfaction with MDTs, length & depth of discussions	Incidence & impact of IT issues on case discussion length, changes in team engagement, quality of interactions, variation in chairing, recording outcomes, and technological satisfaction
Caviola 2023 [30]	Retrospective review, Quantitative	Examine if the pandemic-induced implementation of teleconsultation for Cancer Care Pathway MDTMs supported overall performance and continuity of cancer care delivery	10 CCPs managed by the Quality and Accreditation Office in Reggio Emilia, Italy	10 different cancer types in 20,776 cases	Annual MDT performance using 4 indicators—members’ attendance, number of discussed cases, frequency and duration of meetings	None reported
Perlmutter 2022 [22]	Retrospective observational survey, Quantitative	Identify the keys to success and common pitfalls associated with VMDTMs	20 subspecialty oncology MDTMs at Cleveland Clinic, US	253 Cleveland Clinic health caregivers	Compared in-person vs VMDTs with Likert scale, evaluated ease of participation, common challenges with VMDTMs, potential improvements and further perceptions	None reported
Brims 2021 [29]	Retrospective observational survey, Quantitative	Identify gaps in MDT care and ways to improve service delivery to drive changes in practice	Healthcare institutions treating lung cancer, Australia	Clinicians from 79 separate institutions	Not defined	None reported
Pan 2021 [23]	Observational study (retrospective and prospective), Quantitative	Quantify the impact of a VMSCC on the care quality and patient survival both prospectively and retrospectively with two cohorts	Kaiser Permanente Northern California Integrated Health System, US	141 prospective and 77 retrospective sarcoma cases	Concordance between recommended treatment plan of VMSCC with referring physician	Overall survival and adherence rate to VMSCC treatment plan
Roy 2021 [24]	Prospective observational study, Qualitative	Understanding the underused practice of MDT involvement in radiation therapy treatment planning	Washington University School of Medicine, St Louis, Missouri, US	3 cases for skull-base radiotherapy	Number of cases with changes in aspect of clinical care owing to MDT discussions	None reported
Habermann 2020 [9]	Prospective observational study, Mixed-methods	Evaluate the impact of a MDT lymphoma virtual tumour board	The Mayo Clinic Lymphoma Tumour Board, US	309 confirmed/ suspected lymphoma cases	Not defined	None reported
Dharmarajan 2020 [25]	Retrospective observational survey Mixed-methods	Report the experience of the transition to VMDTMs during Covid-19	Head & neck cancer MDT participants at the University of Pittsburgh, US	19 members of the head and neck MDT	Not defined	None reported
Rosell 2020 [22]	Retrospective observational survey, Mixed-methods	Identify key enabling factors and barriers associated with VMDTMs	National, VMDTMs for rare cancers, Sweden	125 health professionals in 7 national MDTMs for rare cancers	Health professionals' experiences in 3 domains—knowledge and collaboration, decision-making and organizational responsibilities	None reported
Rosell 2019 [27]	Retrospective observational study, Quantitative	Assess participants' views and evaluate their contributions during the meetings	National VMDTMs in Swedish cancer care, specifically for rare cancers	125 health professionals in 7 national MDTM for rare cancers	Not defined	None reported
Amayiri 2017 [31]	Retrospective observational study, Quantitative	Assess the sustainability of a twinned initiative in neuro-oncology between children’s cancer centres and its impact over time	Twinning initiative in neuro-oncology between King Hussein Cancer Centre in Jordan and the Hospital for Sick Children in Canada	> 400 cases from both hospitals	Changes of plans (from pre-conference to post-conference) compared between eras of the VMDT running	Running costs and types of plan change
Montgomery 2016 [26]	Prospective observational study, Quantitative	Demonstrate concordance of consensus real-time diagnosis by local pathologists in Malawi after weekly clinicopathologic teleconferences involving clinicians and pathologists from US and Malawi, and final diagnosis in US to demonstrate success of VMDT model	Weekly clinicopathologic teleconferences involving clinicians and pathologists from the US and Malawi	167 patients evaluated for suspected lymphoma	Concordance between real-time and final decision	None reported
Wilson 2014 [28]	Retrospective observational audit, Quantitative	Audit the impact of the South Australian upper GI MDT against the published guidelines	South Australian upper GI cancer MDT	358 patients were evaluated by upper GI MDT	Proportion of upper GI cases reviewed at MDT; treatment goals generated from MDT recommendation	Time from diagnosis to presentation at MDT, a key performance indicator

*CCP* Cancer care pathway, *GI* Gastrointestinal, *IT* Information technology, *MODe* Metric of decision making, *MOT* Meeting observation tool, *MDT* Multi-disciplinary team, *MDTM* Multi- disciplinary team meeting, *VMDT* Virtual multi-disciplinary team, *VMDTM* Virtual multi-disciplinary team meeting, *VMSCC* Virtual multi-disciplinary team sarcoma case conference, *US* United States

Study designs were highly heterogeneous, although all were observational; specifically, surveys [16, 33, 36, 38, 39, 41, 44], database reviews [34, 37, 40, 42, 43] and a narrative opinion [35]. Eight [33, 35, 36, 38–42] were retrospective, three [16, 35, 37] were prospective and two [34, 44] used both retrospective and prospective methods. Seven studies [33, 34, 37, 39–43] employed quantitative methods, three [16, 36, 44] employed mixed methods, and two [35, 38] used qualitative methods to evaluate the meetings. Two studies [38, 39] reported separate analyses with the same MDT participants: one [39] used a survey and observational tool, while the other [38] only reported on a different survey. One study [35] used a narrative approach to describe the novel use of a video-conference platform. One study [44] incorporated real-time observation, surveys and interviews.

Eight studies [16, 34, 35, 37, 40, 42–44] reported the number of cancer cases discussed during the MDTMs being evaluated, ranging from three [35] to approximately 20,000 cases [42]. Six studies [33, 36, 38, 39, 41, 44] assessed the experiences of members of multiple different MDTs. These studies involved between 19 [36] and 190 [44] health professionals with varying roles.

Survey-based studies [16, 33, 38, 39, 41, 44] primarily involved participants from multiple MDTs, offering broad insights and potential best practices for replication. An exception was one study [36] that surveyed a single MDTM. Only one study [33] compared survey participants’ experiences of in-person versus VMDTMs.

#### Characteristics of evaluated virtual and hybrid MDTMs

Eight studies evaluated vMDTMs [33–36, 38, 39, 41, 42], one evaluated hybrid [16] and one [44] evaluated both virtual and hybrid MDTMs. In three studies, it was not clear if the MDT(s) being evaluated were completely virtual or if there were some face-to-face attendees [37, 40, 43]. Only three studies [35–37] reported the virtual platform used to host the meeting. None of the studies provided a clear definition of virtual or hybrid MDTMs. However, all studies reported the purpose of the MDTM. Nine studies [16, 33, 34, 38–42, 44] were undertaken with specialist or regional MDTs, two [35, 36] with local MDTs, and for two studies, it was unclear if the evaluated MDTs were local or specialist [37, 43].

#### Outcome measures used to evaluate MDT quality and effectiveness

The primary and secondary outcome measures used to assess quality and effectiveness are described in Table 1. Nine studies [33–35, 37, 38, 40, 42–44] clearly defined their outcome measures. Of these, six [34, 35, 37, 40, 43, 44] reported primary outcome measures designed to evaluate changes to clinical diagnosis or treatment plans occurring as a result of the virtual or hybrid MDT discussion. One study [34] examined concordance between the recommended treatment plan from the VMDT sarcoma case conference (VMSCC) with the referring physician, while one [35] assessed the number of cases with changes in clinical care due to MDT discussions. Another study [37] recorded the concordance between real-time diagnoses made by local pathologists in Malawi against the final decision in a weekly clinicopathological teleconference meeting involving clinicians and pathologists from USA and Malawi. A twinning neuro-oncological initiative between children's cancer centres in Jordan and Canada evaluated the changes of plans from pre-conference to post-conference [43]. Of the remaining studies with defined outcome measures, two [33, 38] used survey-derived quantitative and qualitative indicators of MDT members’ experiences and perceptions of quality and effectiveness. Only one study [39] used validated MDT observational tools: the MDT-Meeting Observational Tool (MOT) to assess overall MDT performance; and the MDT-Metric of Decision Making (MDT-MODe) to evaluate each case discussion and participants’ contributions. Scores from these measures were analysed alongside survey data to inform overall conclusions about quality and effectiveness. Another study [44] adopted non-participant MDTMs observations for recording observed IT issues and distractions for individual case discussions, alongside semi-structured interviews on their experiences and views about the impact of COVID-19 on the MDTM.

Five [34, 35, 40, 43, 44] studies reported secondary outcome measures assessing the quality and effectiveness of MDTs. One study [34] recorded the overall survival and adherence rate to the VMSCC treatment plan. Another [43] focused on the running costs and the changes in treatment plans, while Wilson and colleagues [40] used the time from diagnosis to presentation at MDT as a key performance indicator. Roy and colleagues [35] reported numerous secondary outcome measures including MDT members’ perceptions of knowledge, competence, performance, patient outcomes, and team performance. Additionally, another study [44] assessed IT issues on case discussion length, team engagement and quality of interactions, and variability in chairing, recording outcomes, and technological satisfaction, both retrospectively and prospectively.

### Insights into the quality and effectiveness of virtual and hybrid cancer MDTMs

Table 2 reports a summary of key quantitative and qualitative findings from each study relating to MDTM quality and effectiveness. Studies using surveys to capture MDT member experiences and attitudes [16, 33, 36, 38, 39, 41, 44] reported a broad spectrum of views on virtual and hybrid MDTMs. Noted benefits include improvements in clinical care such as number of discussed cases [35, 40, 42], attendance [36, 38, 42, 44], team member knowledge [16, 38], team performance [16, 39], overall survival [34] and patient outcomes [16]. Reported downsides included concerns over discussion quality [33, 44], resource limitations [38, 44] and team cohesion [36, 44].

**Table 2:** Evaluating the effectiveness and quality of virtual and hybrid mdtms: a summary of qualitative and quantitative results

Study	Participants	Quantitative results	Qualitative results
Groothuizen 2023 [32]	190 team members and managers	• Observed 1671 patient discussions across 96 MDTMs, with IT issues recorded in 20.6% of cases, predominantly audio problems • No significant difference in discussion length with IT issues/distractions, except for audio problems, which led to slightly longer discussions	• Five themes identified: attendance and travel time, information sharing, relational and social aspects, communication and participation, and IT issues • Benefits of VMDTMs included increased accessibility and information sharing; limitations involved negative impacts on team engagement and quality of interactions
Caviola 2023 [30]	10 different cancer types in 20,776 cases	• From 2019 to 2022, VMDTs showed overall improvement or no difference in attendance for 9/10 CCPs and for number of cases discussed in 8/10 CCPs over the course of the four years • Observed changes in meeting duration were unrelated to the use of teleconferencing • There was no reduction in the frequency of meetings during the pandemic	N/A
Perlmutter 2022 [22]	253 Cleveland Clinic health caregivers	• Overall, equivalence or superiority between virtual and in-person formats across 9 of 10 participation domains assessed • Improved ease of participating off-site with virtual formats • Small preference for in-person format with quality of discussion	N/A
Brims 2021 [29]	Clinicians from 79 separate institutions	• 93.6% of sites conducted regular MDTMs, but only 57.5% of them achieved the recommended core membership • 69% of sites did not conduct regular audits to ensure compliance with guidelines	N/A
Pan 2021 [23]	141 prospective and 77 retrospective sarcoma cases	• The VMSCC recommended a different treatment plan in 28.2% prospective compared to 19.5% retrospective patients	N/A
Roy 2021 [24]	3 cases for skull-base radiotherapy	N/A	• Significant changes in the clinical target volume delineation for complex radiotherapy cases following virtual discussion of cases
Habermann 2020 [9]	309 lymphoma cases—14 surveys in 2017 and 10 in 2018	• 45% of participants experienced changes in care due to MDT discussions • In both years, 100% of participants rated MDT overall activity as excellent • Overall, 93% of participants perceived improvement in their overall knowledge, competence, performance, and patient outcomes due to VMDTs • Participants stated that tumour board was free of commercial bias; appropriate references and teaching slides were included for educational purposes • No changes to the conference format were recommended	• The MDT improved over time in relation to Continuous Medical Education credit, an in-room microscope, and real-time radiology images
Dharmarajan 2020 [25]	19 members of the head and neck MDT	• 57.9% preferred VMDTs vs 26.3% in-person format. 15.8% had no preference • 31.6% found VMDTs easier vs 31.6% who found in-person format easier. 36.8% found both virtual and in-person formats equally easy • 78.9% preferred VMDTs for future vs 21.1% who preferred in-person format	• Virtual format enhances off-site provider attendance; and reduces the time and burden associated with travel • However, diminished personal interaction and reduced camaraderie among MDT members
Rosell 2019 [27]	125 members in 7 national MDTMs for rare cancers	• Mean scores from the MDT-MOT and MDT-MODe were used to assess VMDTs, resulting in favourable scores for case histories, leadership, and teamwork, whereas patient-centred care and involvement of other care professionals were rated low	
Rosell 2020 [22]	125 health professionals in 7 national MDTMs for rare cancers	N/A	• VMDTs facilitate knowledge sharing, thorough discussion, and competence development. VMDTMs benefit equity and safety but raised concerns about limited availability and consideration of patient factors • Attendance, resource limitations, preparatory work, and variable commitment impact case discussion quality • Resource limitations, uncertainty, and inter-centre competition hinder MDTMs, impeding collaboration and causing misunderstandings
Amayiri 2017 [31]	> 400 cases from both hospitals	• Changes in plans decreased across three consecutive eras over 3 years, decreasing from 44 to 30% and 24% in the respective eras • Reduction in running costs of conferences from $360 to less than $40 per hour	N/A
Montgomery 2016 [26]	167 patients evaluated for suspected lymphoma	4-tier system categorising agreement levels and including the clinical implications of discordance in cytology & Formalin-Fixed Paraffin-Embedded (FFPE) tissue: • Exact match in original vs real-time diagnosis in 74% cytology and 76% FFPE • Similar diagnosis in real-time vs final diagnoses in 9% cytology and 8% FFPE • Change in certainty in real-time vs final diagnosis in 8% cytology and 11% FFPE • Major discordance occurred between 9% cytology and 8% FFPE	N/A
Wilson 2014 [28]	358 patients evaluated by upper GI MDT	• Increase in number and percentage of South Australian Cancer Registry new cases reviewed at MDT between 2010 and 2011 from 43.2% to 54.3% • Patients reviewed within 2 weeks of diagnosis increased from 20 to 50%	N/A

*CCP* Cancer care pathway, *GI* Gastrointestinal, *IT* Information technology, *MDT* Multi-disciplinary team, *MDTM* Multi- disciplinary team meeting, *VMDT* Virtual multi-disciplinary team, *VMDTM* Virtual multi-disciplinary team meeting, *VMSCC* Virtual multi-disciplinary team sarcoma case conference, *US* United States

Perlmutter and colleagues (2022) quantitative survey found that MDT members viewed VMDTMs as comparable to in-person meetings in terms of ease of reviewing pathology and imaging; presenting; gathering subspecialty recommendations and multiple opinions; adding cases; and education [33]. And VMDTMs were rated as superior for facilitating participation in off-site tumour boards. But the quality of case discussions was reported to be better in in-person meetings.

This pattern of increased attendance, but potentially reduced discussion depth, was corroborated by qualitative findings from other studies [36, 38, 44]. Dharmarajan and colleagues (2020) reported that meeting virtually enhances attendance, and reduces the time and burden associated with travel, but diminishes personal interactions and team-working [36]. Conversely, respondents to Rosell and colleagues (2020) survey of members of a national rare cancer MDT found that running MDTMs remotely facilitated greater knowledge exchange and team competence, but this was counterbalanced by instances of inter-site competition and miscommunication [38].

A recent study [44] utilised in-depth interviews and direct observational methods to assess the impact of IT issues on the quality of cancer MDTMs, identifying audio difficulties, which were reported to prolong patient discussions, complicate team interactions and reduce discussion depth and quality.

In addition, the use of standardised observational tools (MDT-MODE and MDT-MOT) in one study to assess VMDT performance provided insights into potential disparities in quality of contribution by professional role and information type in VMDTMs [39]. The MDT-MODE was used to score discussion and contribution quality on a case-by-case basis. Chairs’, surgeons’ and oncologists’ contributions scored highly, with nurses, physiotherapists and MDT co-ordinators receiving the lowest scores. In terms of domains of discussion, the lowest scores were given for contribution of information about the patient view and psychosocial aspect; the highest scoring domain was case history. The MDT-MOT scores meanwhile provided insight into the functioning of the VMDT as a whole. High scores were reported in the domains of clinical decision-making processes; teamworking and culture; technology and equipment; physical environment; and leadership and chairing domains. Whilst patient-centred care, organisation, administration, and post-meeting co-ordination were scored more poorly.

One benefit reported across studies was the ability of VMDTMs to maintain MDTM continuity during the pandemic [42, 44], with the number of members attending and number of cases discussed remaining stable or increasing over the three years since their introduction [42]. VMDTMs were also linked to timely case reviews, with a study noting an increase in cases reviewed within two weeks of referral [40].

Additional findings indicated that VMDTMs contributed to significant changes in treatment plans. Roy and colleagues (2021) reported that VMDT review of three complex cases for skull base radiotherapy led to changes in the target volume delineation compared to a radiation oncologist alone [35]. The study also highlighted the potential educational value of observing or participating in this virtually facilitated process for medical trainees [35]. And Pan and colleagues (2021) review of sarcoma patient treatment plans pre- and post- VMDT discussion, found that VMDT discussion led to treatment plan changes from that of the referring physician in 28.2% (prospective cohort) and 19.5% (retrospective high-grade sarcoma cohort) of cases [34]. Whilst an evaluation of weekly virtual teleconferences involving clinicians and pathologists in the US and Malawi demonstrated 95% concordance rate (i.e. no major discordance) between real-time diagnoses in Malawi achieved through the teleconference; and the final diagnoses in the US following shipping of the case sample [37].

### Facilitators of and barriers to delivery of effective virtual and hybrid cancer MDTMs

Table 3 presents facilitators of and barriers to effective virtual and hybrid cancer MDTMs identified by included studies, grouped into three core categories: (1) IT and infrastructure; (2) Organisation and logistics; and (3) MDT member behaviours and training.

**Table 3:** Facilitators of and barriers to running high-quality and effective virtual and hybrid cancer MDTMs

Facilitators	Barriers
**IT and infrastructure**	
Incorporating video feeds and use of hybrid format [33] Improved interoperability of e-health systems [38] Commercial databases with IT support [41] Reliable teleconference connection and screen sharing capabilities for all members [36]	Limited resources impacting infrastructure and technology [16, 33, 38, 44] Lack of transparency on e-health system compatibility and privacy regulations [35, 38]
**Organisation and logistics**	
Designated time for research protocols, evaluation and feedback of meetings [38, 41] Transparent and accepted referral guidelines [38] Systematic data collection linked to electronic medical record [36] Following the PDSA model for improvement of VMDT setup [36]	Unclear referral guidelines hindering future referrals [39] Irregular meeting dates and scheduling issues [38] Poor time management [16, 35]
**MDT member behaviours and training**	
Reducing travel time to MDTMs promotes member attendance [42, 44] Increased focus on patient-related information with participation from referring physician [38] Open and inclusive meeting atmosphere [38] Clear roles and responsibilities [38] Leadership and teamwork [36] Guidelines for mandatory attendance of key members [38] Development of individual and team competence [36] Complex cases create an exceptional educational environment for participants [16] Brief structured training sessions for chair and participants [35, 38] Dedicated MDT coordinators [41]	Patient-centred care and involvement of nurses and allied healthcare professionals [36] Competition and conflicts between different centres [38] Uncertainty or confusion about roles and assignments [36] Loss of informal conversations during virtual meetings [33, 36, 44] Inadequate methods of communicating treatment recommendations from MDTs [36]

The reported barriers to effective virtual and hybrid MDTMs included challenges with e-health system transparency and resource constraints, such as limited infrastructure and technology, which posed obstacles to integration and meeting effectiveness [16, 33, 38, 41, 44]. The absence of informal discussions and difficulties in conveying treatment recommendations due to networking issues, audio clarity, overlapping conversations, and the inability to see who is speaking undermined the quality of discussions [33, 36, 38, 44]. Particularly notable was the reported deficiency in comprehensive patient-centred discussions and inadequate involvement of care professionals, significantly hindering holistic case evaluations and recommendations [38]. Role uncertainties, time management issues, conflicts between centres, irregular meeting schedules, unclear guidelines, and insufficient emphasis on patient-centred care were also presented as barriers to VMDT effectiveness [35, 38, 41, 43]. Some studies [34, 38] suggested implementing well-defined protocols and clear leadership roles to navigate these challenges.

Other facilitators included having dedicated MDT coordinators, who play a vital role in streamlining virtual meeting organisation [42]. Structured training sessions for chairs and participants was reported to improve meeting efficiency and engagement [41, 43]. Studies found that using VMDTs as a forum to discuss complex cases helped foster an educational environment that improved individual and team competence, ultimately improving team effectiveness [16, 38]. Consistent auditing of the MDT was found to facilitate continuous improvements in VMDT setups [36]. In particular, adopting systematic approaches like the Plan-Do-Study-Act (PDSA) provided a framework for continuous improvement, particularly in transitioning from traditional to VMDT models [36].

Additionally, reliable technology, systematic data collection linked to electronic records, transparent referral guidelines, clear role definitions, and effective leadership were highlighted as being instrumental for optimal VMDT performance [36, 38, 41]. Strategies such as enabling hybrid meeting formats to facilitate reduced travel, and improving e-health system compatibility, enhanced member engagement and overall meeting effectiveness [33, 34, 41, 44].

## Discussion

The review maps the range of diverse settings within which virtual and hybrid cancer MDTMs have been evaluated, and the heterogeneous methods used to do so, predominantly in high-income settings. Key findings indicate that virtual MDTMs improved access to expert consultations, and enhanced efficiency in case discussions, and have facilitated continuity of care during the COVID-19 pandemic. However, challenges were noted in maintaining discussion quality, particularly regarding psychosocial elements and patient-centred care. A notable gap in the literature is the lack of standardized definitions for ‘virtual’ and ‘hybrid’ MDTMs, alongside limited use of validated assessment tools tailored to these formats. The review indicates the need for further research, especially in low-resource settings, to optimize virtual MDTM implementation and address structural and technological barriers.

The review demonstrates that the quality and effectiveness of virtual and hybrid MDTMs have been adopted and evaluated in diverse cancer MDT types, including sarcoma, lymphoma, H&N, CNS, lymphoma, GI and lung, and mixed cancer MDTs. Whilst one study examined use of VMDTs to support access to US experts to discuss diagnostic decisions in Malawi [37], the other studies were all conducted in high-income countries. The utility of the VMDT in Malawi demonstrates the potential of VMDTMs to increase global equity of access to expert care. However, it also highlights a gap in the current literature base and a need for further research into how best VMDTs can be used in this way. For example, explorations of the unique barriers and facilitators to implementing virtual and hybrid MDTMs in lower-income settings, to ensure they can be leveraged to decrease, and not increase, the deprivation gap. Given the evident disparities in cancer care access and outcomes, particularly characterized by lower survival rates, late-stage diagnoses, and reduced treatment access in lower-income and rural areas, VMDTs could be a pivotal solution to help bridge these gaps emphasizing the need for their strategic implementation to truly decrease the global deprivation gap in cancer care [45].

Another gap highlighted by the review is a lack of clear conceptualisation or definition of ‘virtual’ and ‘hybrid’ MDTMs. None of the included studies clearly defined what they meant by these terms; and indeed, numerous different names were used for them including clinicopathologic teleconferences; virtual tumour boards; and VMSCCs. As use of virtual and hybrid MDTMs is likely to continue and potentially increase over time due to a dramatic increase in demand of MDTs [46], it is important to establish a clear and shared understanding of what each of these terms mean. Indeed, in 2013, Munro and Swartzman proposed a basic taxonomy for these terms [29], however, our review identified that in practice these definitions have not been adopted. Given the rapidly evolving technology and cancer care landscape over the past decade, an update to the taxonomy is required to ensure it is up to date, as well as to facilitate its widespread adoption. This would in turn help cultivate usage of standardised terms that could facilitate future systematic evaluations and quality improvement of virtual and hybrid MDTMs.

In line with the diversity of definitions and types of virtual and hybrid MDTMs evaluated by the included studies, a diverse array of study designs and outcomes were used to measure their quality and effectiveness. The current literature on virtual and hybrid MDTMs primarily consists of observational studies, with no randomized experimental designs for direct, gold-standard comparison with face-to-face meetings. Only one study [39] used a validated MDT evaluation tool, even though several tools have been developed to evaluate multiple domains of MDT performance [47]. Importantly though, none of these tools have to date been validated specifically for virtual and hybrid MDTMs. Only one study [44] conducted in-depth qualitative research using interviews and real-time observations, focusing on the impact of IT issues/distractions. Further qualitative work is needed to explore other aspects of VMDT delivery to provide rich, context-specific insights into factors contributing to virtual and hybrid MDT quality and effectiveness [48]. Indeed, the authors of this review have also recently published a study protocol [49] using a mixed-methods approach, combining semi-structured qualitative interviews, a cross-sectional online survey, and live observations to evaluate the quality and effectiveness of VMDTs. This research promises to shed further light on assessing the effectiveness and quality of VMDTMs, with particular emphasis on the group decision-making process, a domain that has not to date been evaluated.

The research identified by this review highlights significant benefits of virtual and hybrid cancer MDTMs, including fostering international collaboration to enhance access to expert care and increasing efficiency related to case handling and review times. These advantages are particularly critical in settings where cancer care pathways face significant strain. For example, in England, the national target for treating patients within 62 days of an urgent referral for suspected cancer has not been met for an extended period [50]. Virtual and hybrid MDT formats allow for more case discussions in less time, potentially mitigating waiting lists and improving access to care. Nevertheless, these formats also pose challenges in preserving thorough discussions, particularly in incorporating psychosocial elements and patient perspectives, which necessitates additional research to preserve the quality of patient-centred decision-making. Effective training and adherence to tailored guidelines are essential to best practice in MDTs. While some guidelines and training for virtual and hybrid MDTs exist, such as the 'Running virtual and hybrid cancer MDTM, an evidence-based toolkit' [51]; developing and evaluating context-specific guidelines for different cancer types and settings may provide further benefits. Additionally, implementing audit processes, such as PDSA cycles [36], is crucial for identifying and addressing issues that affect the delivery of VMDTs, such as IT and infrastructural challenges.

Our findings align with those of Rehman et al. [24], who highlighted the significance of participants' perspectives in assessing the effectiveness and quality of virtual MDTs. However, our scoping review goes further by examining the methodological underpinnings of these evaluations, offering a comprehensive view of how different study designs and outcome measures contribute to our understanding of MDT effectiveness. This methodological focus has unveiled a broader spectrum of evaluations, encompassing not only participant experiences but also the structural, technological, and interactional dimensions of MDTMs.

In this review, we employed a methodological robust approach, adhering to the established Arksey and O’Malley, Levac and colleagues and Joanna Briggs Institute scoping review guidelines [22, 25, 26], and reported in line with the PRISMA-ScR checklist [27]. The screening, charting, analysis and consensus processes involved two reviewers and an interdisciplinary steering committee of behavioural scientists, MDT members, clinicians, service managers and a patient and public involvement representative. Following scoping review guidelines, quality appraisals of included studies were not conducted, and pooled meta-analyses of outcomes were not feasible due to the heterogeneous nature of the study designs and outcomes used. The review therefore does not provide insights into the quality or reliability of the existing evidence base, nor does it provide an objective, quantitative assessment of quality and effectiveness of virtual and hybrid MDTMs. However, the comprehensive mapping of existing literature does provide crucial insights into gaps in the current evidence base, and possible methods for future evaluation, as well as highlighting approaches for clinical teams to consider when implementing and assessing their MDTs. For example, healthcare institutions looking to enhance their VMDT could consider implementing dedicated coordinators, structured training for members and chairs, and clearly defining member roles [35, 38, 41]. Furthermore, the review highlights a need for engagement with policymakers and healthcare managers to address barriers to effective VMDT delivery related to inadequate IT infrastructure, e-health system compatibility and resource limitations [16, 33, 35, 38, 39, 44].

In conclusion, this review has comprehensively mapped existing literature on virtual and hybrid cancer MDTM quality and effectiveness. Current methodological approaches to evaluating virtual and hybrid MDTMs have been spotlighted, and gaps in the current literature identified. In particular, key areas where attention is needed have been highlighted to inform future methodologically rigorous evaluations and ultimately meta-analyses that could quantitatively and definitively compare virtual and hybrid MDTM outcomes to those in face-to-face meetings. This includes a need to establish standardised definitions; validation and use of standardised MDT assessment tools as outcome measures; and use of qualitative research methods to gather rich data on MDT member experience and process. Virtual and hybrid MDTMs offer great potential as an intervention to streamline MDT decision-making and increase equitable access to expert opinion on an international scale, in an era of under-resourcing and stark inequalities in cancer outcomes. However, clear, tailored guidance is required to facilitate their effective delivery, which must be underpinned by the developing evidence base.

## Supplementary Information

Supplementary Material 1

## Data Availability

All data generated or analysed during this study are included in this published article [and its supplementary information files].

## Abbreviations

CCP: Cancer care pathway
CNS: Central nervous system
FFPE: Formalin-fixed paraffin-embedded
GI: Gastrointestinal
HCP: Healthcare professional
H&N: Head and neck
IT: Information technology
MDT: Multi-disciplinary team
MODe: Metric of decision making
MOT: Meeting observation tool
MDTM: Multi-disciplinary team meeting
PDSA: Plan-do-study-act
PRISMA-ScR: Preferred reporting items for systematic reviews and meta-analyses for protocols and scoping reviews
RCT: Randomised controlled trial
SPIDER: Sample, phenomenon of interest, design, evaluation, research type
SMG: Study management group
US: United States
VMDT: Virtual multi-disciplinary team
VMDTM: Virtual multi-disciplinary team meeting
VMSCC: virtual multi-disciplinary team sarcoma case conference
